# Hepatoprotective effect of syringin combined with costunolide against LPS-induced acute liver injury in L-02 cells via Rac1/AKT/NF-κB signaling pathway

**DOI:** 10.18632/aging.205161

**Published:** 2023-11-01

**Authors:** Jingxin Mao, Lihong Tan, Cheng Tian, Wenxiang Wang, Hao Zhang, Zhaojing Zhu, Yan Li

**Affiliations:** 1 Chongqing Medical and Pharmaceutical College, Chongqing 400030, China; 2 College of Pharmaceutical Sciences, Southwest University, Chongqing 400715, China; 3 Chongqing Key Laboratory of High Active Traditional Chinese Drug Delivery System, Chongqing 400030, China; 4 Chongqing Three Gorges Medical College, Chongqing 404120, China

**Keywords:** *Dolomiaea souliei* (Franch.) C.Shih, syringin, costunolide, acute liver injury, hepatoprotective effect

## Abstract

Acute liver injury (ALI) leads to abnormal liver function and damage to liver cells. Syringin (syr) and costunolide (cos) are the major extracts from *Dolomiaea souliei* (Franch.) C.Shih (*D. souliei*), showing diverse biological functions in various biological processes. We explored the underlying hepatoprotective effects of syr+cos against LPS-induced ALI. Cell viability and proliferation were assessed using an MTT assay and immunofluorescence staining. Flow cytometry analysis was used to detect cell cycle distribution and apoptosis. ELISA was utilized to measure liver function and antioxidant stress indexes. qRT-PCR and western blotting was performed to determine mRNA and protein levels respectively. Using shRNA approach to Rac1 analyzed transcriptional targets. The results showed that syr+cos promoted L-02 cell proliferation, inhibiting the cell apoptosis and blocking cell cycle in G1 and G2/M phase. Syr+cos decreased the production of ALT, AST, LDH, MDA and ROS while increased SOD and CAT activities. Pretreated with syr+cos may decrease expressions of caspase-3,7,9, NF-κB, TNF-α proteins, Cyclin B, CDK1 and p-IκB proteins while p-IκB increased. Silencing of Rac-1 may protect the liver by increasing AKT, S473, T308 and reducing p-AKT proteins. Syr+cos exhibits anti-ALI activity via Rac1/AKT/NF-κB signaling pathway which might act as an effective candidate drug for the treatment of ALI.

## INTRODUCTION

The liver’s significance in the synthetic and metabolic activities of the organism is essential, but it can be impaired by various chemicals [1]. Some cyanogenetic chemicals together with lipopolysaccharide (LPS) and carbon tetrachloride (CCl_4_) might cause acute liver injury (ALI), and there are established experimental cell models for evaluating the hepatoprotective activities of medicines [2, 3]. When kupffer cells (KCs) are stimulated by LPS, they begin to release the pro-inflammatory cytokine including the tumor necrosis factor-alpha (TNF-α) and interleukin (IL)-6, that are central and important factors to the development of ALI [4].

Liver injury, liver dysfunction or liver pathology is recognized as a heavy pathological state that causes the 5^th^ high mortality worldwide [5]. ALI is a serious disease (clinical syndrome) that can cause massive necrosis or apoptosis of hepatocytes, steatosis of hepatocytes, inflammatory reaction, oxidative stress, and liver function damage, often resulting in high mortality [6]. With the continued deterioration of hepatocyte function, liver fibrosis, cirrhosis, and eventually liver failure ensue. The advanced stage of ALI is a severe condition that currently lacks any specific cure apart from undergoing a liver transplant [7]. Although enormous progress has been made to cure different liver disorders, the progress in treating individuals with ALI is not optimal [8]. Hence, there is an imminent need to create new and effective hepatoprotective agents/drugs that hold promise for treating ALI in a clinical setting.

Despite the significant mortality rates associated with ALI, effective therapies are still lacking in recent years. Currently, some of the most popular phytoconstituents with hepatoprotective properties include flavonolignans such as silymarin, lignans like schizandrin, and triterpenoids such as glycyrrhizin [9–11]. However, above of three drugs might cause varies adverse reactions or further injured on organs [12]. Therefore, there is a pressing need for medication that is highly effective, has a clear mechanism of action, and low incidence of side effects. Natural products are valuable sources for developing new drugs to treat liver diseases, particularly those that are inflammatory-induced ALI in nature [13]. *D. souliei* has been utilized as a traditional folk medicine for centuries to assuaged pain and treat gastrointestinal ailments in the Western Sichuan and Eastern Tibet regions of China [14]. Earlier studies have established that sesquiterpene lactones, sesquiterpene lactone dimers (SLDs), lignans, and triterpenes are the principal bioactive compounds found in *D. souliei* [15, 16]. There’re barely clinical efficacy of *D. souliei* but focus is on *in vivo* or *in vivo*. Upon being extracted from *D. souliei*, these above related compounds demonstrate a series of pharmacological effects, including anti-bacterial (*in vivo*) [17], anti-tumor (*in vitro*) [18], anti-inflammatory (*in vivo*) [19], and anti-oxidant activities (*in vitro*) [20].

Syringin (syr) is regarded as a prominent precursor of lignans, which are isolated from the roots of *D. souliei* in the ethyl acetate (EA) soluble fraction. This substance has garnered growing interest due to its ability to inhibit inflammation and regulate immune responses [21], as well as its potential to treat conditions like DalN/LPS-induced fulminant hepatic failure [22] and alleviate acute lung injury caused by LPS [23]. In addition, syr was reported to exhibit anti-hyperglycemic activity (*in vitro*) [24], anti-fatigue effect (*in vivo*) [25], useful for releasing acetylcholine, increasing insulin secretion (*in vitro*) [26] and preventing cardiac hypertrophy and diabetic cardiomyopathy (*in vitro*) [27, 28]. Costunolide (cos) is a natural sesquiterpene lactone with anti-cancer (*in vivo*) [29], anti-oxidant (*in vivo*) [30], anti-inflammatory (*in vivo*) [31], neuroprotective (*in vitro*) [32], and anti-diabetes (*in vivo*) [33] properties. In recent years, it was reported that cos also exhibits the anti-liver injury property [34]. Pilot studies have demonstrated the multiple pharmacological properties of syr or cos alone, but the specific role of syr+cos in hepatoprotection and the mechanisms involved are still unclear. To address this gap, we investigated the latent effects of syr+cos against ALI and to ascertained the hepatoprotective mechanisms against LPS-induced damage to L-02 hepatocytes.

## MATERIALS AND METHODS

### Materials and reagents

In September of 2015, *D. souliei* was accumulated from Sichuan Province of China and subsequently identified and confirmed as authentic by Professor Chen in room 315 at Southwest University of China using a voucher specimen/sample (NO. CMX2017-014) [35]. The LPS was provided by Shanghai Lianzu Biotechnology Co., Ltd. (Shanghai, China) for this study. The total protein extraction kit, MTT assay and RIPA lysis buffer were obtained from AmyJet Scientific Inc. (Wuhan, China). Primary antibodies for rabbit Cyclin B, CDK1, NF-κB (p65 nucleus/cytosol), Caspase 3,7,9, p-AKT (S473), p-AKT (T308), IκB, p-IκB, and IKK α/β, Tublin, β-actin, mouse TNF-α, and secondary antibodies (IgG-HRP-conjugated) were purchased from Wuhan Fine Biotech Co., Ltd. (Wuhan, China), Thermo Fisher Scientific Inc. (Shanghai, China) and Beyotime Biotechnology Co., Ltd. (Shanghai, China) respectively. Additionally, GenePharma Co., Ltd. (Shanghai, China) supplied the Rac1 short hairpin RNA (shRNA) plasmid (Rac1 group) and empty plasmid vector (control group).

### Extraction and isolation

**Figure 1:**
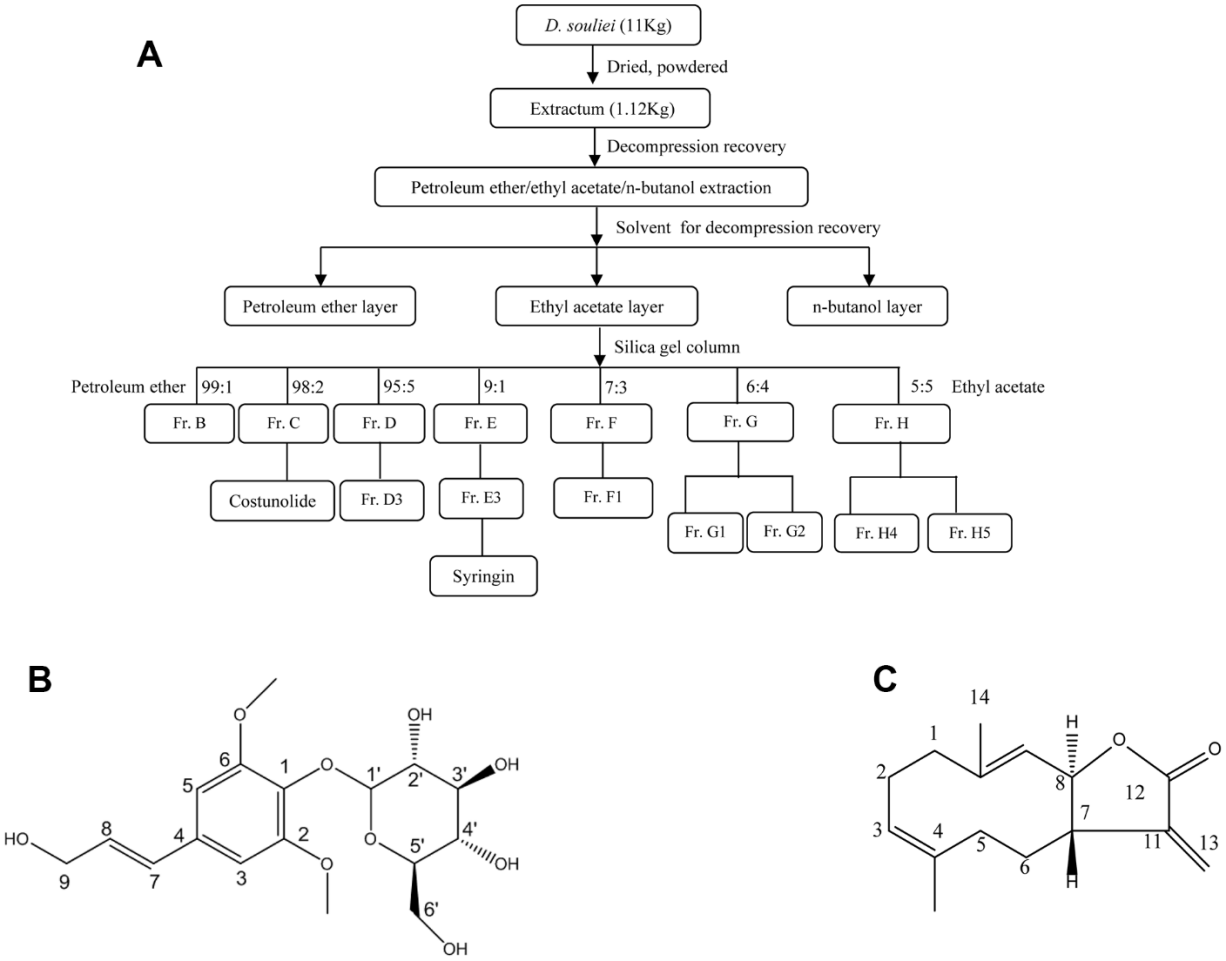
(**A**) The separation process diagram of syr+cos that derived from *D. soulei* and (**B**) chemical structure of syr. (**C**) Chemical structure of cos.

After air-drying, 11.0 kg of the roots of *D. souliei* was processed into a powder and then extracted using 95% ethanol via maceration at 37° C overnight, then evaporated under vacuum, partitioned and extracted with petroleum ether, ethyl acetate, and n-butanol sequentially. 296 g of a residue from petroleum ether part was finally obtained from a total of 14 fractions. Among above fractions, syr (20 mg, molecular formula: C_17_H_24_O_9_) and cos (20 mg, molecular formula: C_15_H_20_O_2_), which were crystallized and recrystallized from fraction C and fraction E respectively (Figure 1A). Syr with the purity of 99.09%, which was confirmed using spectrographic method of ^1^H-NMR (Supplementary Figure 1) and ^13^C-NMR (Supplementary Figure 2) respectively by comparing with previous literature [36]. Cos with the purity of 99.19%, was confirmed using spectrographic method of ^1^H-NMR (Supplementary Figure 3) and ^13^C-NMR (Supplementary Figure 4) respectively by comparing with previous literature [37]. Furthermore, the reference fingerprint and HPLC fingerprints of *D. souliei* were presented in Supplementary Figures 5, 6 respectively indicating syr and cos are the main compounds from *D. souliei*. Syr, white powder, molecular weight: 372.37, melting point: 174-177° C. Cos, white to almost white powder to crystal, molecular weight: 232.32, melting point: 106-107° C. The chemical property of compounds syr and cos was presented in Supplementary Files 1, 2 respectively.

### Cell culture and syr+cos treatment

The L-02 cells were obtained from the Cell Bank of Typical Culture Preservation Committee (CNTCPC) at the Chinese Academy of Sciences (Beijing, China). They were then cultured in RPMI-1640 medium containing 10% fetal bovine serum, 100 units/mL penicillin, and 100 μg/mL streptomycin, and maintained in a humidified environment with 95% air and 5% CO_2_ at 37° C. To induce acute liver injury and establish ALI model, LPS was dissolved in RPMI 1640 medium with concentration of 60 μg/mL, which was added to the L-02 cells after 24 hours of culture. Adherent cells were detached using ethylene diamine tetraacetic acid (EDTA) and plated onto 96-well plates at 70-80% confluence. Syr (molecular formula: C_17_H_24_O_9_, relative molecular mass: 327.37) and cos (molecular formula: C_15_H_20_O_2_, relative molecular mass: 232.32) were obtained from the root of *D. souliei* and then dissolved in DMSO. To treat LPS-induced L-02 cells, syr+cos was administered at final concentrations of either 10 or 40 μM, with DMSO being used as the control. The concentration of dimethyl sulfoxide (DMSO) added was adjusted to ensure it did not exceed 0.1%. All experiments were conducted independently in triplicate.

### MTT cell viability assay

The 3-(4,-dimethylthiazol-2-y)-2,5- diphenyl-tetrazolium bromide (MTT) assay was utilized to evaluate the cell viability (Sigma Aldrich, St. Louis, MO, USA). L-02 cells in their logarithmic growth phase, with a density of approximately 70-80%, were seeded onto 96-well plates initially. These were then allowed to adherent growth overnight. Subsequently, different concentrations of syr+cos (10 and 40 μM) as well as 40 μM silymarin (sily) were added to LPS (60 μg/mL) induced L-02 cells (with cell density approaching 70-80%) and were cultured for a duration of 48 hours. DMSO was utilized as the control. At specified time points/intervals (1-7 days), the L-02 cells were treated with MTT (5 mg/mL, 20 μL per well) and incubated for 4 hours at 37° C. After incubation, formazan crystals were dissolved in DMSO (150 μL) at room temperature, and the absorbance was measured at 560 nm using a microplate reader (Bio-Rad 550; Bio-Rad Laboratories, Inc., Hercules, CA, USA). The analysis of data was carried out utilizing GraphPad Prism 8.0 (GraphPad Software, Inc., La Jolla, CA, USA). All experiments were conducted independently in triplicate.

### BrdU staining

To monitor cell proliferation, BrdU staining was performed. Initially, 1×10^6^ L-02 cells were seeded onto 24-well plates and were adherent growth overnight in a 37° C incubator. 40 μM of syr+cos was then added to the medium, with DMSO being used as the control. After 48 hours, 10 μg/mL of BrdU (Sigma Aldrich, USA) was added to the cells for a duration of 2 hours, followed by fixation with 4% paraformaldehyde for 15 minutes. The cells were then treated with 2 mol/L HCl and 0.3% TritonX-100, followed by washing with phosphate-buffered saline (PBS) three times for 5 minutes. The cells were blocked with 10% goat serum for 1 hour at room temperature (Beyotime Biotechnology Inc., Shanghai, China). Subsequently, the cells were incubated with BrdU primary antibody (dilution 1:500, Sigma Aldrich, St. Louis, MO, USA) at 4° C overnight, and washed with PBS three times for 5 minutes. The cells were then incubated with BrdU secondary antibody (dilution 1: 200, Sigma Aldrich, St. Louis, MO, USA) for a duration of 2 hours. The fluorescent signals were observed under a fluorescent microscope (Leica DMIL, Wetzlar, Germany), and BrdU-positive cells in random fields were counted. Relative fluorescence quantitative analysis was determined using ImagePro 6.0 software.

### Flow cytometry analysis

Cells were cultured in medium supplemented with 5 μM demethylzeylasteral and were harvested for flow cytometry analysis, with DMSO being used as the control. For the cell cycle assay, cells treated with syr were collected after 48 hours, washed with cold PBS, fixed in 75% ethanol at 4° C for 48 hours, and then incubated in a PBS solution containing 1 μL PI (BD, San Jose, CA, USA) and 1 μL RNaseA (Sigma Aldrich, USA) at 37° C for 30 minutes. BD accuri C6 flow cytometry (BD, USA) was then used to analyze cell cycle changes. Conversely, for the cell apoptosis assay, cells were treated with demethylzeylasteral and then collected after 48 hours, washed twice with cold PBS, and incubated in 100 μL of binding buffer (BD, USA) containing PI (5 μL) and AnnexinV-APC (BD, USA, 2.5 μL) at room temperature for 20 minutes. Flow cytometry, along with FlowJo software, was used to analyze the cell cycle and apoptosis of L-02 cells. All experiments were independently performed in triplicate.

### ALT, AST and LDH assay

L-02 cells were incubated in 6-well microtiter plates at a density of 1×10^6^ cells/well per well and treated with 60 μg/mL LPS for 4 hours before being treated with syr for 48 hours. The cells were washed three times with PBS and the culture medium was discarded. To collect the treated cells, 100 μL phosphate-buffered saline (PBS) was added followed by lysis with 100 μL of Triton-100 cell lysis buffer (Beyotime Institute of Biotechnology, Shanghai, China). Uniform collection of L-02 hepatocytes was achieved through full mixing and blowing of the lysate. After centrifugation at 3000 rpm for 10 minutes at 4° C, commercially available kits (Nanjing Jiancheng Bioengineering Research Institute, Nanjing, China) were employed to measure the levels of ALT, AST, and LDH, as per the manufacturer’s instructions, respectively.

### MDA, CAT and SOD assay

Biochemical analysis was conducted by assessing the levels of MDA, CAT, and SOD in the cell culture supernatant using commercial kits procured from the Institute of Biological Engineering of Nanjing Jiancheng (Nanjing, China) and Beyotime Institute of Biotechnology (Shanghai, China) for their respective assays.

### ROS assay

To quantify intracellular ROS levels, the DCFH-DA/H2DCFDA (2’,7’-dichlorofluorescein diacetate)-cell reactive oxygen species detection kit (Abcam, Cambridge, UK) was utilized. DCFDA was oxidized by ROS in viable cells to form 2’,7’-dichlorofluorescein (DCF), which is highly fluorescent at 529 nm. The cells were washed 3 times with PBS before adding DCFDA diluted to a final concentration of 20 μM. The cells were then incubated for 45 min at 37° C in the dark. After washing with PBS three times, fluorescence was measured with a multimode microplate reader (Tecan Trading AG, Männedorf, Switzerland) at excitation and emission wavelengths of 495 nm and 529 nm, respectively. The ROS level was calculated as the absorbance ratio between the experimental cells and the control cells and expressed as a percentage.

In order to measure the levels of intracellular ROS, the DCFH-DA/H2DCFDA (2’,7’-dichlorofluorescein diacetate)-cell detection kit (Wuhan Chemstan Biotechnology Co., Ltd., Wuhan, China) for ROS was employed. The viable cells oxidized DCFDA with ROS to produce 2’,7’-dichlorofluorescein (DCF), which emits highly fluorescent light at 529 nm. Prior to adding the DCFDA diluted to a final concentration of 20 μM, the cells were washed 3 times with PBS and then incubated for 45 min at 37° C in the dark. Following three additional washes with PBS, the fluorescence was measured using a multimode microplate reader (Thermo Fisher Scientific Inc., Waltham, MA, USA) with excitation and emission wavelengths of 495 nm and 529 nm, respectively. The absorbance ratio between the experimental cells and control cells was used to calculate the percentage of ROS level respectively.

### Construction of pLKO.1-shGFP and pLKO.1-Rac1 vector

Sangon Biotech Company in Shanghai, China designed and synthesized primers for the Rac1 gene based on the coding sequence (CDS) region of the gene in Genbank (Table 1). LPS-induced L-02 cells were seeded at a density of 1 × 10^5^ cells/well in 24-well plates and pretreated with syr+cos when the cell density reached 70~80%. Following the washing of cells with cold PBS, total RNA was extracted from them using TRIzol (Invitrogen, USA) in accordance with the manufacturer’s instructions (n=5). The Rac1 gene was amplified in triplicate using the extracted total RNA as a template through reverse transcription polymerase chain reaction (RT-PCR). The amplified products, including pLKO.1, were subjected to agarose gel electrophoresis and EB staining for detection (5 μL). After recovery and purification, the amplified products were digested with BamH and EcoR digestion enzymes. The Rac1 fragment was then ligated to the pLKO.1 carrier using T4 ligase after gel recovery. The competent DH-5alpha was transformed and inoculated in a culture dish coated with ampicillin at 37° C for 14 hours. The colonies were selected, inoculated in liquid culture medium containing ampicillin, and cultured for 12 hours. The plasmid underwent a series of procedures, including extraction, digestion using BamH I, EcoR I, and Hind III, and subsequent analysis by agarose gel electrophoresis, polymerase chain reaction (PCR), and sequence analysis. Finally, the recombinant plasmid containing the Rac1 gene was successfully constructed.

**Table 1:** Primer sequence design of Rac1.

**Primers**	**Sequence**
Forward primer of Rac1	5′-AAGCTAGGATCCCAGGCCATCAAGTGTGTG-3′
Reverse primer of Rac1	5′-AGGCGCCGAATTCTTACAACAGACGGCATTT-3′

### Plasmid transfection and shRNA interference

L-02 cells induced by LPS were cultured on a 24-well plate, and when the cell density reached approximately 70~80%, the pLKO.1-Rac1 vector was added to serum-free 1640 medium and mixed for 15 minutes before being cultured in full medium for 6 hours. Three shRNA sequences targeting the Rac1 gene in L-02 cells were designed using the online software BLOCK-iT™ RNAi Designer (https://rnaidesigner.thermofisher.com/rnaiexpress/design.do), ThermoFisher’s RNAi website) and are listed in Table 2. The cells were transfected using lipofectamine 3000 transfection reagent according to the manufacturer’s instructions. RNA and protein were extracted 48 hours after transfection, and the shRNA with the highest transfection efficiency was selected for subsequent experiments.

**Table 2:** Sequence design of shRNA.

**Number of shRNA**	**Sequence**
shRNA#1	CGCAAACAGATGTGTTCTTAA
shRNA#2	GCTAAGGAGATTGGTGCTGTA
shRNA#3	CCTTCTTAACATCACTGTCTT

### Total RNA extraction and real-time fluorescence quantitative PCR

We isolated total RNA from LPS-induced L-02 cells transfected with pLKO.1-Rac1 using Trizol reagent (Invitrogen, Carlsbad, CA, USA) according to the manufacturer’s instructions. The expression level of Rac1 mRNA was then quantified using the SYBR Premix Ex Taq™ II kit (Takara, China) for quantitative reverse transcription PCR (qRT-PCR). We used tubulin mRNA levels for normalization, and the specific primer pairs are listed in Table 3. We calculated the relative expression of Rac1 mRNA compared to tubulin mRNA using the 2^-ΔCT^ method.

**Table 3:** Primer sequence design.

**Primers**	**Sequence**
Forward primer of Rac1	5′-GTAAAACCTGCCTGCTCATCA-3′
Reverse primer of Rac1	5′-GGACGCAATTCATAATCTTC-3′
Forward primer of Tublin	5′-ATTCAACGGCACAGTCAAGG-3′
Reverse primer of Tublin	5′-GCAGAAGGGGCCGGAGATGA-3′

### Western blot analysis

After collection, cells with a density of approximately 70~80% were lysed in RIPA lysis buffer supplemented with phenylmethyl sulfonyl fluoride. The cell lysates were denatured at 100° C for 30 minutes, and the resulting extracts were centrifuged at 12000 g at 4° C for 10 minutes. The protein samples were then separated on a 10% and 12% SDS-PAGE gel respectively at 100 V and transferred onto nitrocellulose membranes (Beyotime Institute of Biotechnology, Shanghai, China) using a semi-dry transfer system. The blots were blocked for 2 hours at room temperature in 10% skim milk and then incubated overnight at 4° C with primary antibodies dissolved in 10% fetal calf serum PBS. The primary antibodies used for western blotting were rabbit CDK1 (1:1000), Cyclin B (1:1000), Caspase 3,7,9 (1:1000), NF-κB p65 (1:1000), TNF-α (1:1000), Rac1 (1:1000), AKT (1:1000), p-AKT (1:1000), IκB (1:1000), p-IκB (1:1000), IKK α/β (1:1000), p-AKT (S473) (1:1000), p-AKT (T308) (1:1000) Tublin (1:1000) and β-actin. The secondary antibody used was goat anti-rabbit (1:2000) (Beyotime Institute of Biotechnology, Shanghai, China) at room temperature for 1 hour, and the blots were washed with PBST for 5 minutes, repeated 3 times. Finally, the target proteins were visualized and quantitated using the ECL system and Image Jet software (Amersham, Buckinghamshire, UK) with Tublin as the internal standard.

### Molecular docking verification

The 2D structures of syr and cos were obtained from the PubChem database (https://pubchem.ncbi.nlm.nih.gov) and saved in “SDF” format. The 3D structure of the protein corresponding to the core target, which includes CDK1, Cyclin B, Caspase 3, 7, 9, NF-κB, and TNF-α, was downloaded from the PDB database (https://www.rcsb.org) and saved in “PDB” format. Chem3D 14.0 software and PyMOL 2.5 software were utilized to remove the original ligands and water molecules and add hydrogen atoms, respectively. Molecular docking was performed between the core target protein receptor and the small molecule ligands of syr and cos using Discovery Studio 2019 software. The binding activity was evaluated using the LibDock score, and Discovery Studio 2019 software was used to draw the binding mode diagram between the core target protein receptor and the small molecule ligand of syr and cos. The resulting diagrams were displayed in both 3D and 2D structures.

### Statistical analysis

Triplicate samples and data were collected and analyzed. Mean standard deviation (SD) and statistical significance were calculated using Excel (Microsoft, Albuquerque, NM, USA) and SPSS 20.0 (IBM, Armonk, NY, USA). The results were presented as mean ± SD for the three replicates and analyzed using one-way ANOVA followed by Dunnett’s multiple comparison tests. *P*-values less than 0.05 and 0.01 were considered significant and very significant, respectively.

### Data sharing statement

All the data that support the findings of the study are included in the article or available from the corresponding author, upon reasonable request after publication.

## RESULTS

### Effects of syr+cos on cell viability

**Figure 2:**
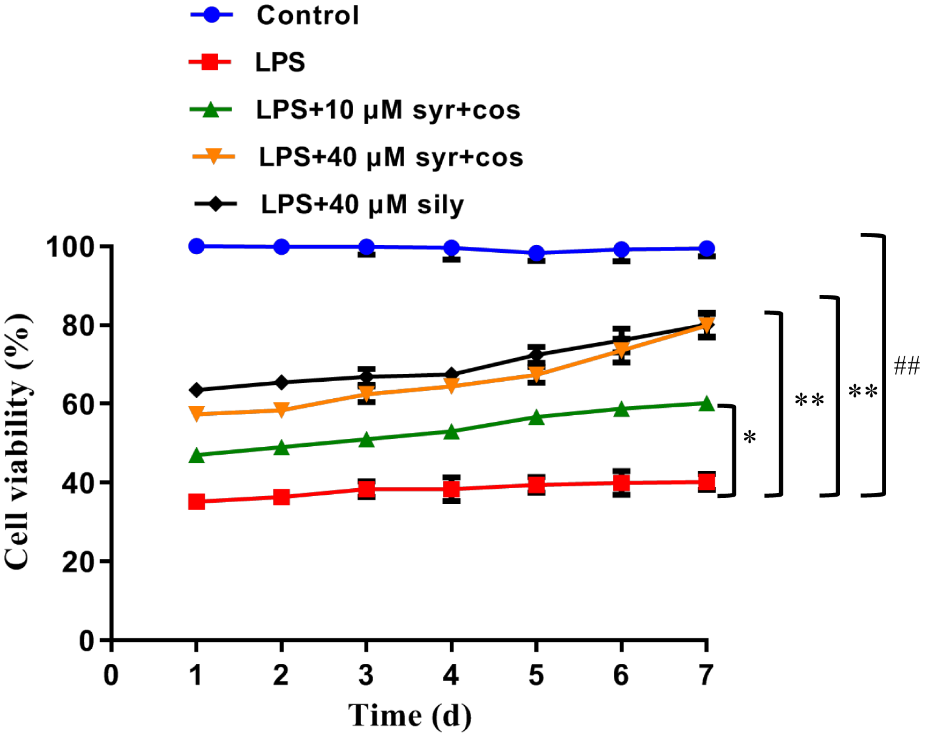
**Effects of syr+cos on the cell viability of L-02 cells.** Note: The data represent the mean ± SD per group. ^**^P < 0.01 or ^*^P < 0.05 vs control group; ^##^*P* < 0.01 vs LPS treatment group.

Syr is a kind of phenylpropanol glycosides compound, and its chemical structure was shown in Figure 1B. Cos is a kind of sesquiterpenoid compound, and its chemical structure was shown in Figure 1C. To determine whether syr+cos could protect LPS-induced L-02 cells against ALI. L-02 cells were exposed to LPS at 60 μg/mL concentration while syr+cos at 10 μM and 40 μM respectively administrated for consecutive 7 days. As shown in Figure 2, the cell viability of L-02 cells was decreased significantly (*P* < 0.01) in LPS treatment group compared with control group. However, the cell viability were significantly increased at concentration of 10 μM (*P* < 0.05) and 40 μM (*P* < 0.01) respectively while pretreated with syr+cos compared with LPS treatment group. As well as the sily treatment group (*P* < 0.01). Therefore, the growth of L-02 cells were significantly promoted with syr+cos *in vitro*. Furthermore, cell proliferation was more obvious in 40 μM syr+cos treatment group compared with 10 μM syr+cos treatment group.

### Effects of syr+cos on cell proliferation

**Figure 3:**
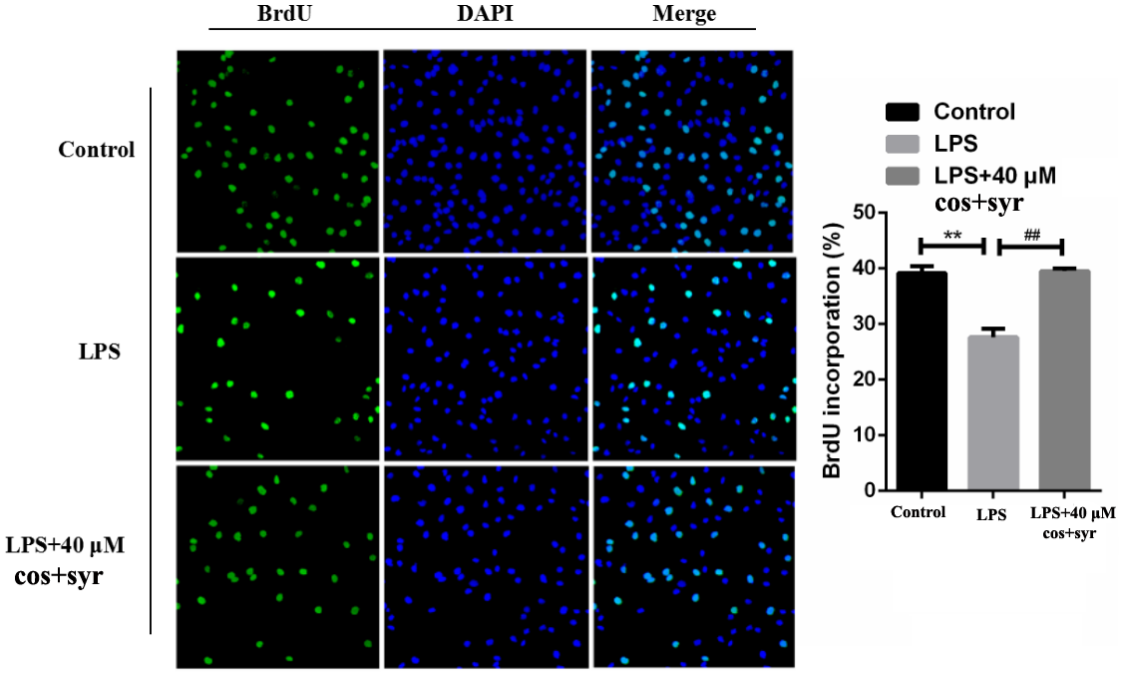
**Effects of syr+cos on L-02 cell proliferation by BrdU immunofluorescence detection (100×).** Note: The data represent the mean ± SD per group. ^**^P < 0.01 vs control group; ^##^*P* < 0.01 vs LPS treatment group.

In the DNA synthesis phase (S phase), BrdU can replace thymine and infiltrate into the replicated DNA molecule, and then stained with fluorescent labeled BrdU antibody, thus the cell proliferation can be detected [38]. Using BrdU method to investigate the effects of syr+cos on the proliferation of L-02 cells. Compared with the control group, immunofluorescence microscopy result revealed that the quantity of L-02 cell staining was decreased in the nucleus of cells with LPS treatment group. Nevertheless, the quantity of L-02 cell staining was increased in the nucleus of cells treated with 40 μM syr+cos compared to LPS treatment group (Figure 3). Therefore, the proliferation of L-02 cells was markedly promoted while pretreated with syr+cos.

### Effects of syr+cos on cell cycle

**Figure 4:**
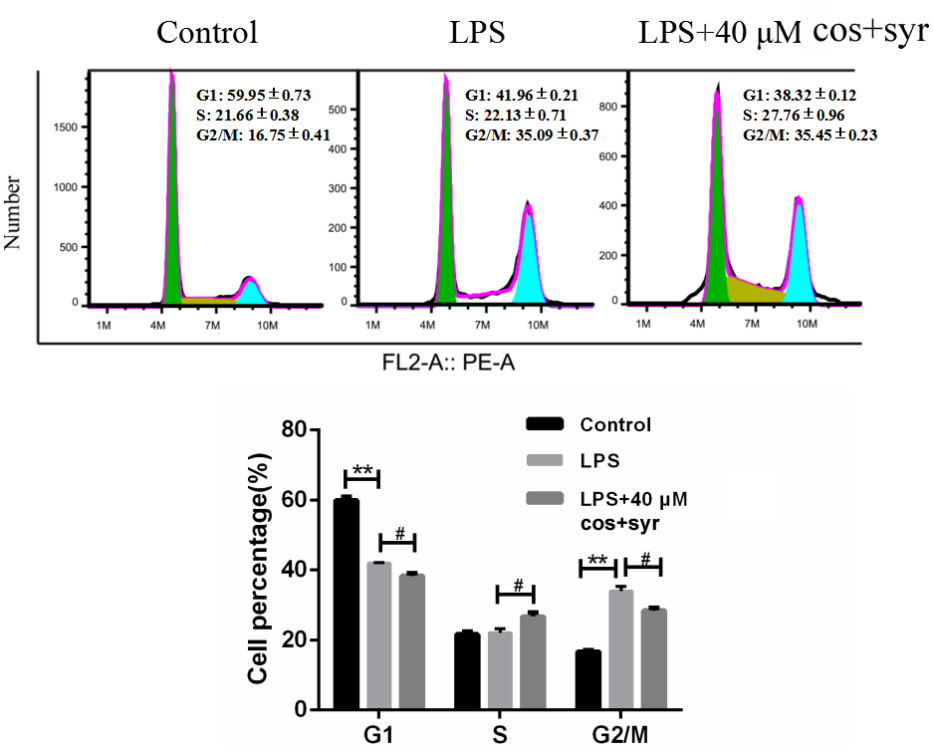
**Effects of syr+cos on cell cycle in L-02 cells.** Note: The data represent the mean ± SD per group. ^**^P < 0.01 vs control group; ^#^*P* < 0.05 vs LPS treatment group.

The cell cycle process is regulated by several molecules through distinct mechanisms in numerous diseases [39]. To assess the impact of syr+cos on the cell cycle progression of L-02 cells, PI staining was performed. After treating the cells with syr+cos for 48 hours, their cell cycle distribution was analyzed by flow cytometry, as illustrated in Figure 4. Compared with control group, the proportion of G_2_/M phase cells in LPS treatment group increased from 16.94 ±0.09% to 35.27 ±0.23%. Compared with LPS treatment group, the percentage of G_2_/M phase cells in 40 μM syr+cos treatment group decreased from 35.27 ±0.11% to 28.80 ±0.56%, the proportion of S phase cells increased from 21.53 ±0.36% to 25.99 ±0.41%, and the proportion of G_1_ phase cells decreased from 40.24 ±0.82% to 37.10 ±0.57%. It was proved that syr+cos has an hepatoprotective effect against ALI on the cell cycle of LPS-induced L-02 cells by blocking in G_1_ and G_2_/M phase.

### Effects of syr+cos on cell apoptosis

**Figure 5:**
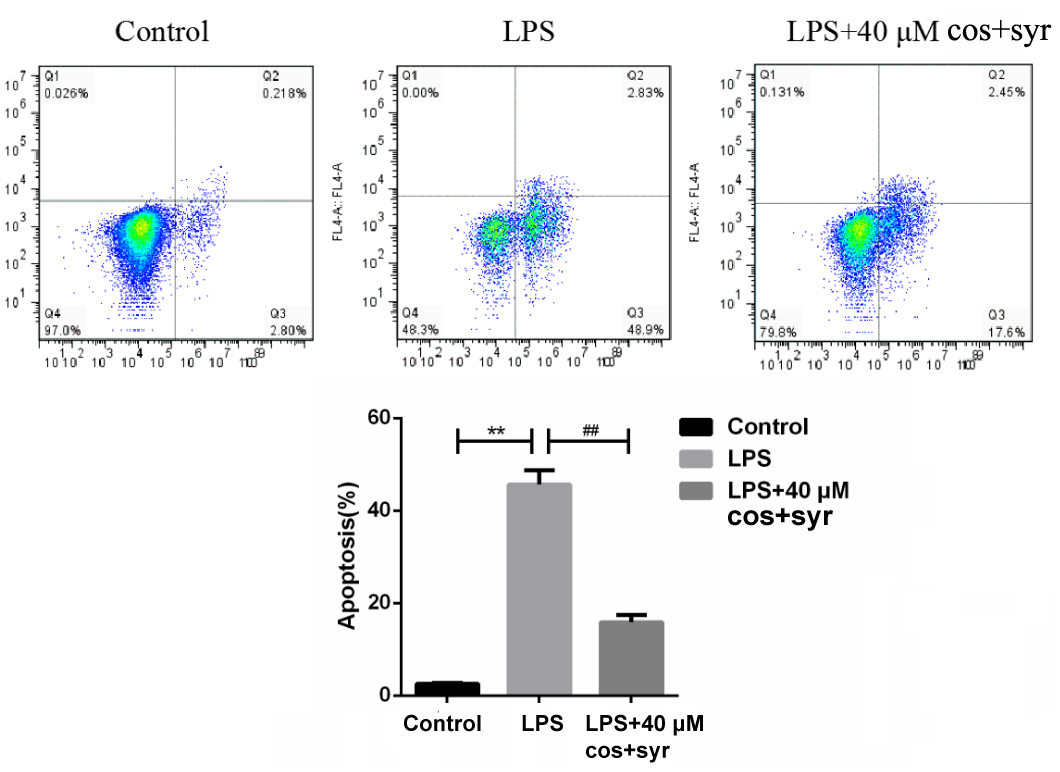
**The results of annexin V and PI double staining after syr+cos treatment (L-02 cells were treated with 40 μM for 48 h, the apoptotic cells were quantified by flow cytometry).** Note: The data represent the mean ± SD per group. ^**^P < 0.01 vs control group; ^##^*P* < 0.01 vs LPS treatment group.

Apoptosis may be a basic biological development of cells, which plays a vital role in removing unwanted or abnormal cells in cellular organism [40]. The various populations of cells could also be discovered once cells area unit double stained with annexin V [41]. The apoptosis rate of L-02 cells significantly decreased from 51.73 ±0.11% (LPS treatment group) to 20.05 ±0.23% (40 μM syr+cos group) after 48 h syr+cos administration (Figure 5). It was revealed that syr+cos administration may significantly reduce the apoptosis of LPS-induced L-02 cells, thus protecting L-02 cells from ALI.

### Effects of syr+cos on liver function and biochemical measurement

**Figure 6:**
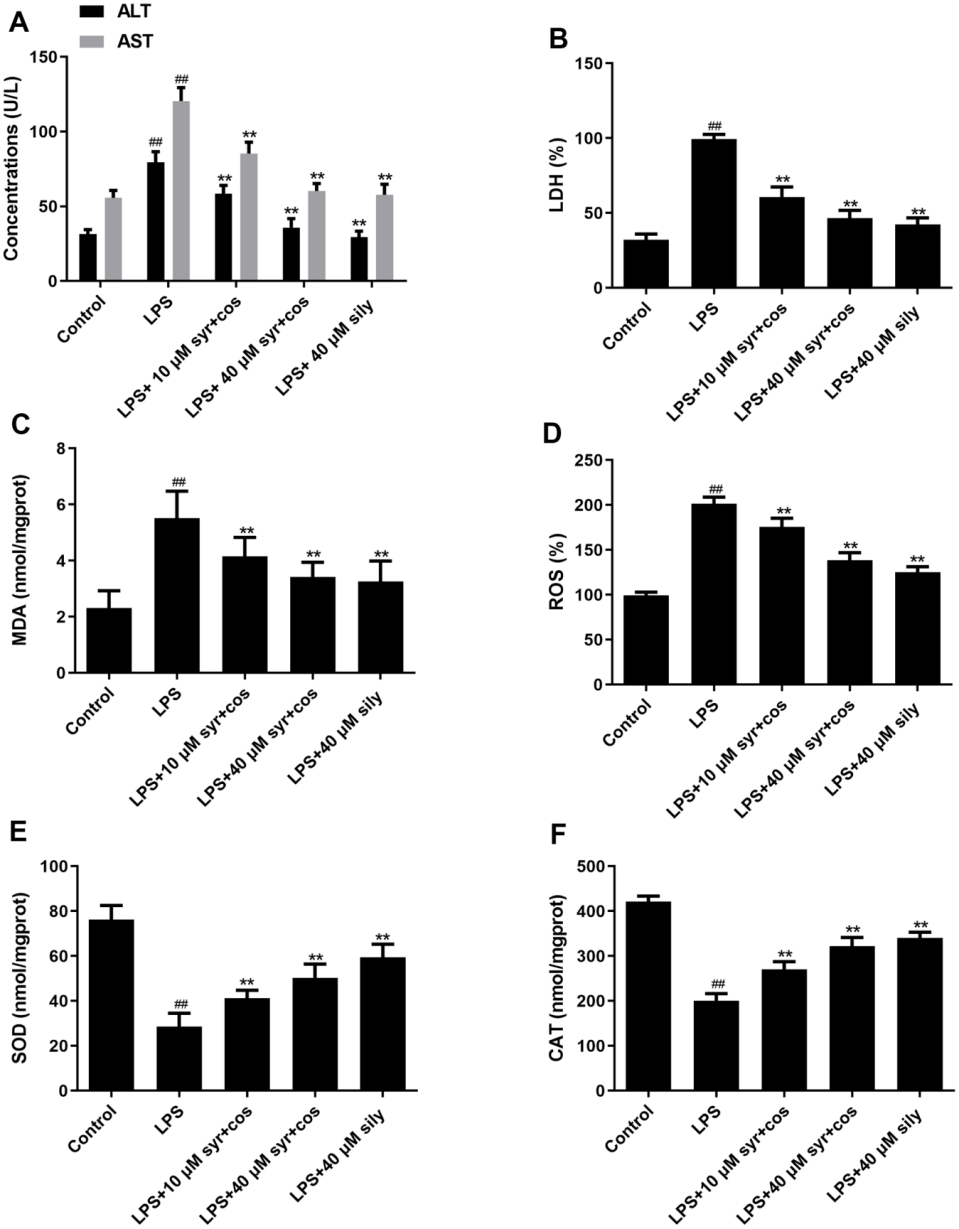
Effects of syr+cos on liver function (**A**) ALT, AST, (**B**) LDH and indicators related to oxidative stress (**C**) MDA, (**D**) ROS, (**E**) SOD and (**F**) CAT respectively. Note: The data represent the mean ± SD (n = 5) per group. ^**^P < 0.01 vs control group; ^##^*P* < 0.01 vs LPS treatment group.

To further assess the hepatoprotective activity of syr+cos, the release of ALT, AST, and LDH into the culture medium was measured. Compared with control group, ALT, AST and LDH levels were markedly improved in LPS-induced group (Figure 6). Compared with LPS-induced group, ALT, AST (Figure 6A) and LDH (Figure 6B) levels were markedly suppressed when administrated with syr+cos. The levels of MDA (Figure 6C), ROS (Figure 6D), SOD (Figure 6E), and CAT (Figure 6F) activities were confirmed by using appropriate ELISA kits respectively. The levels of SOD, CAT were reduced while increased in MDA and ROS in LPS-induced group. However, administrated with varies concentration of syr+cos which may reverse this trend. The data indicated syr+cos exhibits the potent antioxidant potential and hepatoprotective activity.

### Effects of the syr+cos on the expression levels of NF-κB, TNF-α, caspases-3, 7, 9, Cyclin B, CDK1, IKK α/β, IκB, p-IκB, and p65 (nucleus or cytosol)

**Figure 7:**
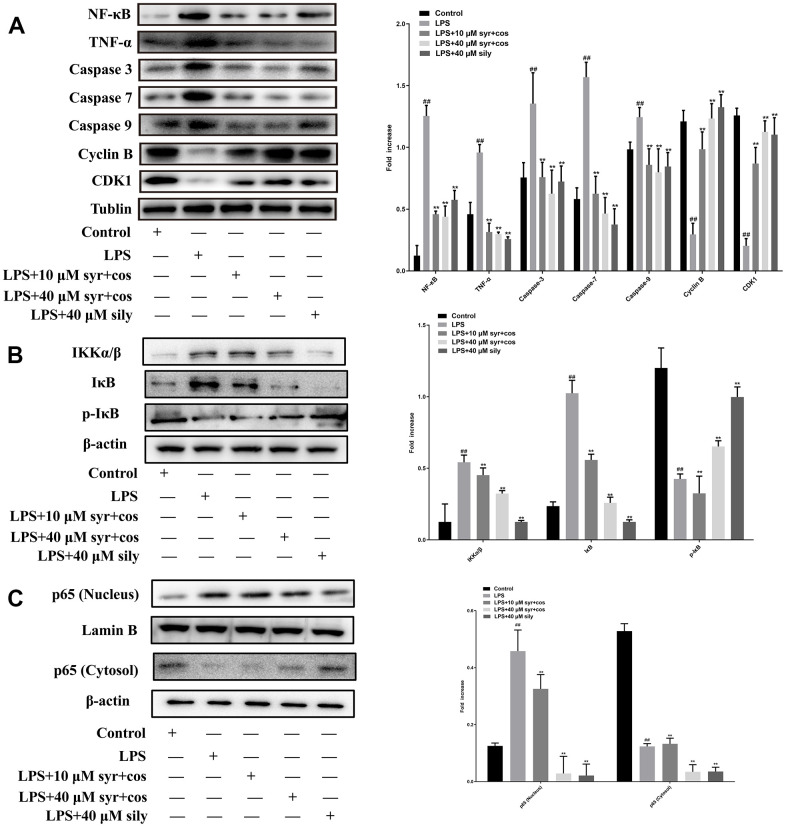
(**A**) Effects of syr+cos on inflammatory pathway, cell cycle and apoptosis-related proteins respectively. (**B**) Effects of syr+cos on NF-κB related signaling pathway. (**C**) Effects of syr+cos on p65 (nucleus) and p65 (cytosol). Note: The data represent the mean ± SD (n = 5) per group. ^**^P < 0.01 vs control group; ^##^*P* < 0.01 vs LPS treatment group.

The expression levels of NF-κB, TNF-α, caspases-3, 7, 9, Cyclin B and CDK1 proteins were presented (Figure 7A). The expression levels of IKK α/β, IκB, p-IκB proteins were displayed in Figure 7B. The expression levels of p65 (nucleus or cytosol) proteins were shown in Figure 7C.

TNF-α is considered the most crucial pro-inflammatory cytokine in the pathology of ALI. TNF-α is an activator of the NF-κB signaling pathway, and the release of NF-κB amplifies the pro-inflammatory response cascade which finally lead severe inflammation occurred [42, 43]. Compared with the control group, the expression of NF-κB and TNF-α proteins was markedly increased in LPS treatment group (^**^P<0.01). Compared with the LPS treatment group, the expression level of NF-κB and TNF-α proteins was markedly reduced in syr+cos treatment group (^##^*P*<0.01). The results revealed that syr+cos might exhibit hepatoprotective effect against LPS-induced L-02 hepatocytes ALI by down-regulating the expression of NF-κB and TNF-α proteins respectively.

Caspases, a family of cysteine proteases that are conserved through evolution, which play a momentous role in the inflammatory and cell death responses in ALI [44]. This family comprises both initiator caspases (e.g. caspases 8, 9, and 10) and effector caspases (e.g. caspases 3, 6, and 7) [45]. Compared with control group, the expression of caspase-3, 7, 9 proteins was markedly expanded in LPS treatment group (^**^P<0.01). Compared with the LPS treatment group, the expression level of caspase-3, 7, 9 proteins was significantly decreased in syr+cos treatment group (^##^*P*<0.01). The results revealed that syr+cos could protect LPS-induced L-02 hepatocytes ALI by inhibiting the expression of apoptosis proteins (caspase-3, 7, 9).

Cyclin B binds to CDK1, forming the cyclin B-CDK1 complex, which is required for entry into mitosis and progression through the cell cycle [46]. Generally speaking, Cyclin B is considered to be the principal cyclin protein which may regulate CDK1 activity during the transition from phases G_2_ to phases M [47]. CDK1 kinase mainly regulates G_2_/M phase, when the content of Cyclin B accumulates to a certain value, Cyclin B and CDK1 bind to each other to form a complex, CDK1 makes substrate protein phosphorylation, and leads to chromosome condensation, nuclear fibronectin phosphorylation, and finally disintegration of nuclear membrane [48]. Compared with the control group, the expression of Cyclin B and CDK1 proteins was markedly reduced in LPS treatment group (^**^P<0.01). Compared with the LPS treatment group, the expression level of Cyclin B and CDK1 proteins was markedly increased in syr+cos treatment group (^##^*P*<0.01). The results showed that syr+cos could protect LPS-induced L-02 hepatocytes ALI by promoting the expression of Cyclin B and CDK1.

NF-κB as the most important nuclear transcription factor in cells, is involved in the transmission of many intracellular signaling pathways and the transcription and regulation of genetic information [49]. Its signaling pathways mainly include activation of IκB kinase, IκB protein degradation and nuclear transport of p65. It was reported that the NF-κB signaling pathway was activated when the IKK complex (IKKα, IKKβ and IKKγ) was activated by an upstream signal [50]. Then, IKK complex especially IKKα/β could be degraded by ubiquitination and promote p65 phosphorylation. Therefore, we detect the protein expression level of IκB, p-IκB, and IKK α/β, as well as the changes in the p65 (nucleus or cytosol) protein level [51]. IκB kinase, causing IκB phosphorylation and degradation (proteasome dependent ubiquitination degradation), release of p65 and p50 heterodimers, exposure of nuclear localization sequences, and rapid entry into the nucleus. In the present study, it was found that compared with the control group, the expression of IκB and IKK α/β proteins was markedly increased while p-IκB reduced in LPS treatment group (^**^P<0.01). Compared with the LPS treatment group, the expression level of IκB and IKK α/β proteins was significantly reduced while p-IκB increased in syr+cos treatment group (^##^*P*<0.01). The results showed that syr+cos could protect LPS-induced L-02 hepatocytes ALI by attenuating expression of IκB and IKK α/β and promoting the expression of p-IκB. In addition, the western blot analysis result of cytosol and nucleus p65 proteins showed that the p65 protein translocated from cytosol to the nucleus after administrated with LPS. However, cos+syr administration may significantly reduce this translocation.

### Results of molecular docking

**Figure 8:**
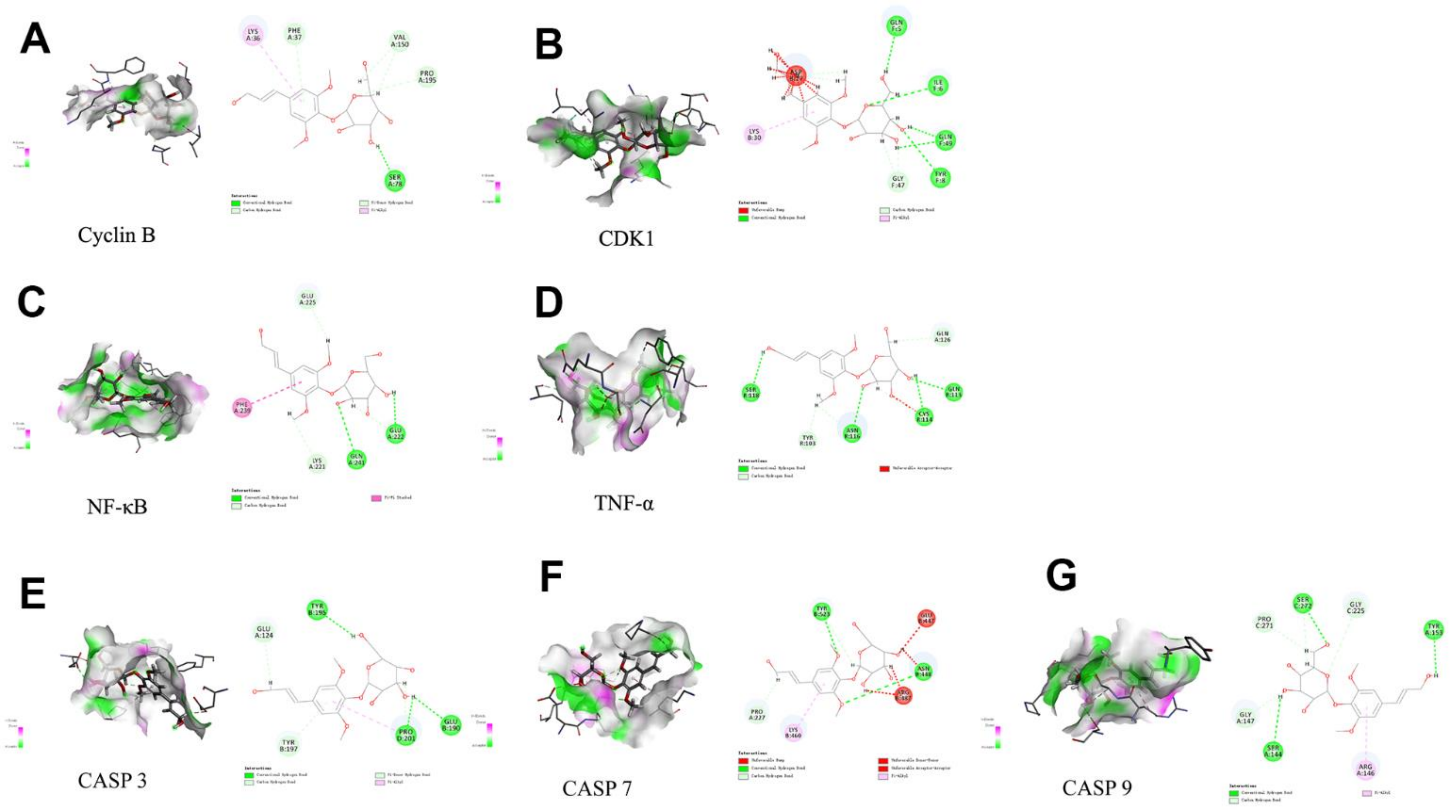
**Molecular docking of syr with 7 proteins.** (**A**) Cyclin B, (**B**) CDK1, (**C**) TNF-α, (**D**) NF-κB, (**E**) CASP3, (**F**) CASP7, and (**G**) CASP9 respectively.

**Figure 9:**
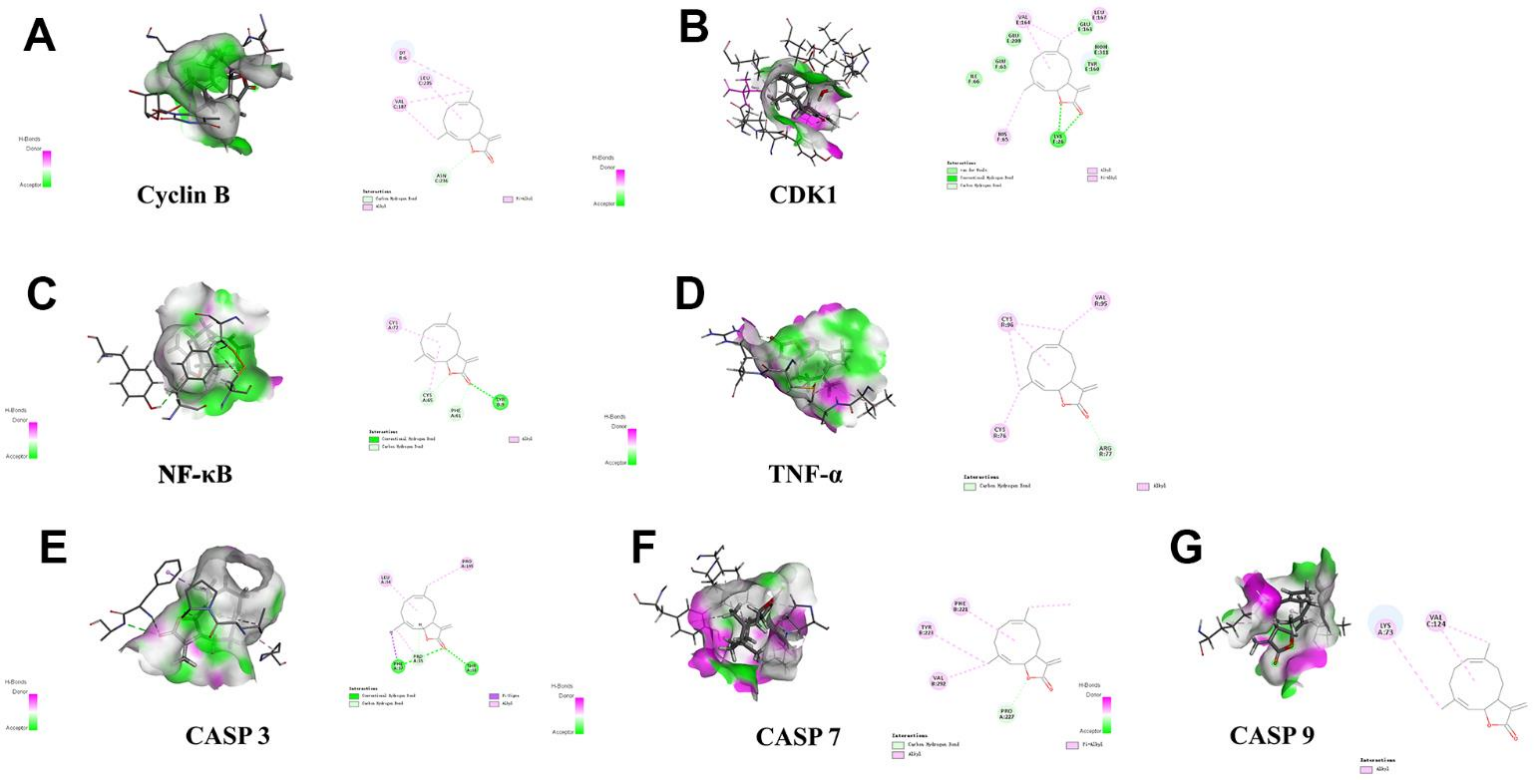
**Molecular docking of cos with 7 proteins.** (**A**) Cyclin B, (**B**) CDK1, (**C**) TNF-α, (**D**) NF-κB, (**E**) CASP3, (**F**) CASP7, and (**G**) CASP9 respectively.

Discovery Studio 2019 software was utilized to examine active ingredients and highly connected targets identified through the application of network pharmacology. Molecular docking visualization results indicated that syr+cos effectively entered the active site of the key target protein, and interacted with specific amino acid residues through hydrogen bonding and protein interactions. Among them, syr forms 4 hydrogen bonds with the PHE37, VAL150, PRO195, and SER78 residues of Cyclin B protein (Figure 8A); 5 hydrogen bonds were formed with the GLN5, ILE6, GLN49, TYR8, and GLY47 residues of CDK1 protein (Figure 8B); 4 hydrogen bonds were formed with GLU225, GLU222, GLN241, LYS221 residues of NF-κB protein (Figure 8C); 6 hydrogen bonds were formed with SER118, TYR103, ASN116, CYS114, GLN113, and GLN126 residues of TNF-α protein (Figure 8D); 5 hydrogen bonds were formed with GLU124, TYR195, TYR197, PRO201, and GLU190 residues of Caspase 3 protein (Figure 8E), 3 hydrogen bonds were formed with TYR523, ASN448, and PRO227 residues of Caspase 7 protein (Figure 8F); 6 hydrogen bonds were formed with the PRO271, SER272, GLY225, TYR153, SER144, and GLY147 residues of Caspase 9 protein (Figure 8G). The molecular function of those binding sites may influence the stability of binding of compound. Cos forms 1 carbon hydrogen bond (ASN236) and 3 alkyl (DT6, LEU235, VAL187) residues of Cyclin B protein (Figure 9A); 1 hydrogen bond (LYS26) and 3 alkyl (HIS65, VAL164, GLU163) were formed with the residues of CDK1 protein (Figure 9B); 1 hydrogen bond (TYR9), 1 alkyl (CYS72) and 2 carbon hydrogen bond (CYS65, PHE61) were formed with residues of NF-κB protein (Figure 9C); 3 alkyl (CYS76, CYS96, VAL95) and 1 carbon hydrogen bond (ARG77) were formed with residues of TNF-α protein (Figure 9D); 2 hydrogen bond (THR38, PHE37), 2 alkyl (PRO195, LEU34) and 1 carbon hydrogen bond (PRO35) were formed with residues of CASP3 protein (Figure 9E); 3 alkyl (PHE221, TYR223, VAL292) and 1 carbon hydrogen bond (PRO227) were formed with residues of CASP7 protein (Figure 9F); 2 alkyl (LYS73, VAL124) was formed with residues of CASP9 protein (Figure 9G). The biological activity of a protein typically depends on the presence of a small number of functional residues (e.g. above amino acid residues combined with compounds in different ways or competitive activation vs deactivation with the molecule substrates). Although residues are predicted to be functional, conservation patterns are often more complicated. In addition, the hydrogen bond lengths formed between the compound and the key target are all 1-3, which indicating that the hydrogen bond distance between amino acids and the compound is relatively close and the binding is relatively tight. All of the core targets, syr had the strong binding ability (presented as LibDock score) with Cyclin B (133.704), CASP 7 (90.1649), NF-κB (98.5878), CASP 9 (65.4471), TNF-α (108.556), CDK1 (95.7877), CASP 3 (86.8322) respectively. Cos had the strong binding ability with Cyclin B (70.359), CASP 7 (958.214), NF-κB (73.6911), CASP 9 (71.5776), TNF-α (68.4741), CDK1 (68.4288), CASP 3 (85.3545) respectively (Table 4).

**Table 4:** The results of molecular docking.

**Compound**	**Target**	**PDB**	**LibDock score**
syringin	Cyclin B	1bu2	133.704
syringin	CASP 7	1f1j	90.1649
syringin	NF-κB	1ikn	98.5878
syringin	CASP 9	1jxq	65.4471
syringin	TNF-α	1tnr	108.556
syringin	CDK1	4yc6	95.7877
syringin	CASP 3	7seo	86.8322
costunolide	Cyclin B	1bu2	70.359
costunolide	CASP 7	1f1j	58.214
costunolide	NF-κB	1ikn	73.6911
costunolide	CASP 9	1jxq	71.5776
costunolide	TNF-α	1tnr	68.4741
costunolide	CDK1	4yc6	68.4288
costunolide	CASP 3	7seo	85.3545

### The efficiency of RNAi silencing Racl gene expression

**Figure 10:**
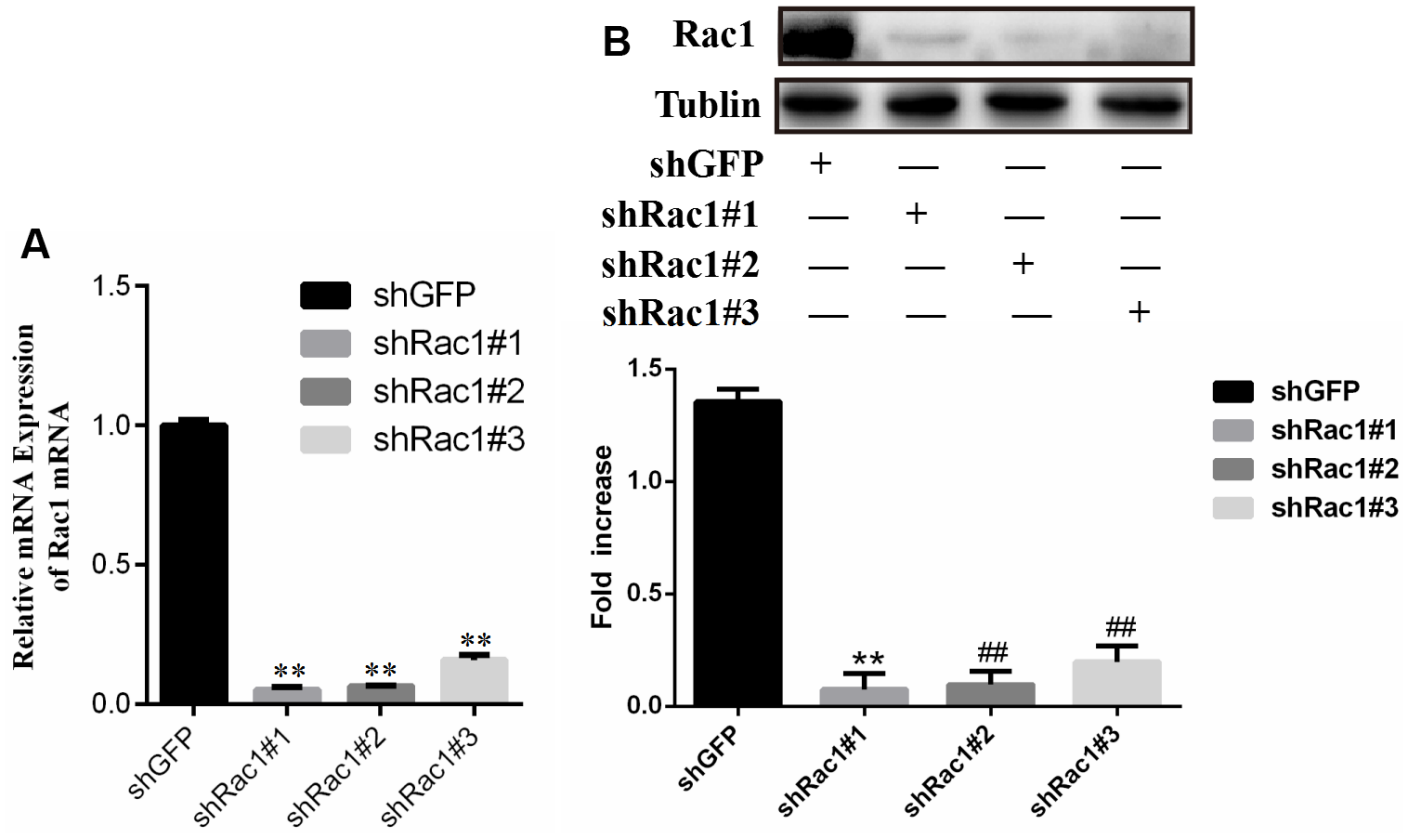
Comparison of Rac1 gene silencing efficiency of shRac1#1, shRac1#2 and shRac1#3 construct for (**A**) PCR result and (**B**) western blot result respectively.

L-02 cells were exposed to Lentivirus containing Racl shRNA interference plasmid or GFP negative control plasmid, and incubated for 48 hours. The qRT-PCR and western blot analyses were utilized to assess the mRNA (Figure 10A) and protein levels (Figure 10B) of Racl respectively. Results indicated a significant reduction in Racl expression in the shRacl#1, shRacl#2, and shRac1#3 groups compared to the shGFP control group, indicating effective interference. The group with the highest efficiency of interference was identified as shRacl#1 and selected for subsequent experiments (Figure 10).

### Expression of Rac1/AKT signaling pathway after rac1 gene silencing

**Figure 11:**
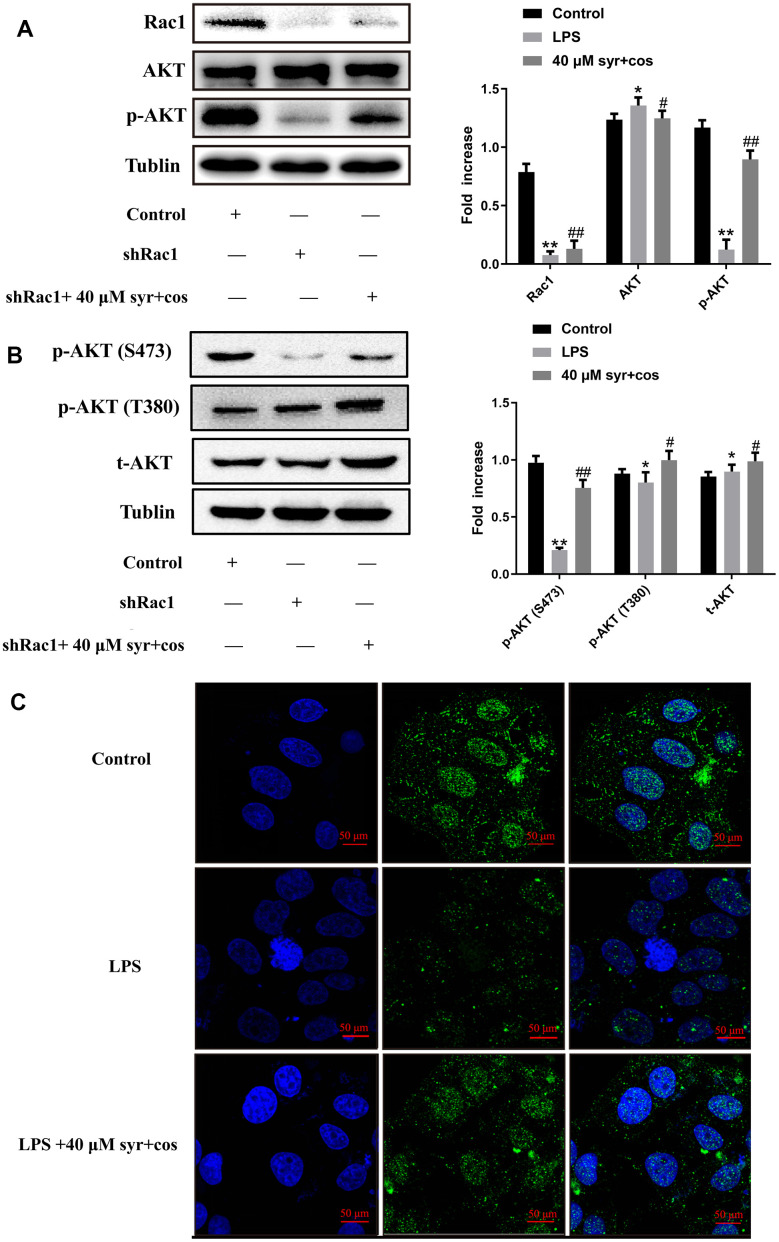
(**A**) The expression of Rac1, AKT, p-AKT proteins respectively. (**B**) The expression of IKK α/β and IκB, p-IκB proteins respectively. (**C**) The BrdU immunofluorescence results of p-AKT (100×).

Previous research suggests that changes in Racl protein expression may lead to the phosphorylation of AKT protein in cells [52]. In this study, shRNA was utilized to silence Racl gene expression in L-02 cells, and protein expression of Racl, AKT, and p-AKT were evaluated through western blot. Results indicated a significant enhancement in AKT protein expression, and a reduction in p-AKT protein expression, in the Racl interference group compared to control. However, treatment with 40 μM syr+cos can reverse this trend significantly (Figure 11A). PI3K is a phosphatidylinositol 3-kinase generated by the binding of extracellular receptors and corresponding ligands, which can promote the formation of a second messenger phosphatidylinositol (3,4,5) triphosphate (PIP3) [53]. After phosphorylation by T308 and S473, it can fully activate the kinase (AKT), a key downstream of PI3K, serine/threonine. AKT is generally believed to be activated on the plasma membrane, and after activation, it enters the cytoplasm or nucleus [54]. The S473 site disrupts the hydrophobicity of AKT, which fully activates AKT activity. After exercising their function, T308 and S473 are dephosphorylated to terminate AKT signaling [55]. Therefore, in order to confirm whether the drug activates AKT, we used western blot analysis to detect the phosphorylation level of AKT Thr308/Ser473 site following reviewer’s suggestion. The specific results are shown in Figure 11B. The result revealed that a significant reduce in S473 and T308 protein expression, and an increase in t-AKT protein expression, in the Racl interference group compared to control. However, treatment with 40 μM syr+cos may increase the expression of S473, T308 and t-AKT proteins significantly. In addition, the condition of the nucleus was observed through cellular immunofluorescence staining, while the expression of p-AKT was verified through fluorescence staining. DAPI staining is shown in Figure 11C, and there is no bright blue fluorescence in the control group, indicating normal liver cells and no nuclear apoptosis. The LPS group showed dense and dense staining of the liver nuclei with bright blue fluorescence, indicating that LPS induced a large number of liver cell apoptosis. Compared to LPS group, LPS+40 μM syr+cos group was able to significantly reduce LPS induced liver cell apoptosis. The fluorescence staining results are consistent with western blot results. This indicates that syr+cos may significantly increase the expression level of p-AKT protein, reduce the rate of nuclear apoptosis, and thus achieve a protective effect against LPS induced L-02 cell damage.

## DISCUSSION

Liver injury can be caused by various substances or factors, such as viruses, alcohol, drugs, toxins, and hypoxia, that directly damage hepatocytes [56]. It may lead to pathological changes, which including inflammation, fibrosis, apoptosis, and necrosis of hepatocytes, finally resulting in liver dysfunction and various liver diseases [57]. The repair and regeneration process after liver injury is generally accomplished through the coordinated efforts of hepatocytes and KCs [58]. To better understand the related genes and signaling pathways involved in liver repair, it is crucial to clarify the mechanisms and pathways of genes involved [59]. Analysis of key node genes in the literature suggests that apoptosis, cell cycle, and inflammation might play significant roles in the development and progression of hepatocyte injuries [60, 61].

Previous studies reported that syr could protect hepatocytes from injury by promoting hepatocyte proliferation and immune-related ways [62]. It was also reported that cos may remarkably suppress the proliferation of HepG_2_ hepatocellular carcinoma cells and inhibited autophagy [63]. In order to further investigate the hepatoprotective effect of syr+cos against LPS-induced ALI in L-02 cells, the proliferation, cell cycle and apoptosis of L-02 cells were detected. It was revealed that the repair process of liver is complex, which including the ability of resting hepatocytes to enter the cell cycle induced by cytokines in the initiation phase, and the ability of proliferating cells to proliferate through continuous division (through G_1_, S, G_2_ and M phases) until the recovery of liver function [64]. It has also been reported that about 95% of the resting phase (G_0_ phase) hepatocytes will synchronously enter the cell cycle through proliferation when liver injury occurs, thus increasing the overall number of hepatocytes [65]. Based on the above reports, BrdU fluorescence staining was used to reflect cell proliferation. The results indicated that compared with LPS treatment group, the amount of BrdU-positive cells in syr+cos treatment group increased by 10%. It was proved that syr+cos administration could proliferate L-02 cells. In addition, the cell cycle distribution of L-02 cells was detected by flow cytometry PI staining. The results revealed that the cells in G_1_ and G_2_/M phases of L-02 cells were gradually decreased after syr+cos administration, while the cells in G_1_ and G_2_/M phase were gradually increased indicating that syr+cos could block the cell cycle in G_1_ phase and G_2_/M phase respectively. Previous research has demonstrated that drugs can promote the proliferation and regeneration of hepatic parenchymal cells, thereby preventing cell necrosis or apoptosis after liver injury and ultimately restoring liver function by replacing necrotic or apoptotic hepatocytes [66]. Our research supports this finding, as it was discovered that allicin notably inhibits L-02 cell apoptosis and reduces AST and ALT levels, ultimately providing protection to L-02 cells [67]. In the present experiment, Annexin V/PI result showed that syr+cos could significantly inhibit L-02 cell apoptosis at 48 hours after treatment, thus achieving the protection of L-02 cells, which was basically consistent with Chen’s research.

Apoptosis, programmed cell death controlled by genes to maintain intracellular homeostasis, is associated with the pathogenesis of numerous liver diseases, including fulminant hepatic failure, hepatic injury, viral hepatitis, hepatic fibrosis, cirrhosis, and hepatocellular carcinoma [68]. The caspase family of proteins is integral in inducing and executing apoptosis, with caspase-3 playing a vital role in caspase-mediated cell death [69]. Caspase-7 also acts as a mediator of apoptosis by amplifying the cascade reaction during the apoptosis process [70]. Upon receiving a signal, cytochrome c firstly binds to apaf-1 then to activate caspase-9, which transmits apoptotic information to caspase-3 to trigger the apoptotic response [71]. Additionally, caspase-3 and -7 have been found to play a role in regulating immune homeostasis, with their expression being up-regulated following LPS stimulation but down-regulated after H_2_O_2_ treatment [72]. Our study found that syr+cos protects L-02 hepatocytes from LPS-induced injury by inhibiting the expression of apoptotic proteins, including caspase-3, 7, and 9, as shown by western blot analysis.

Cyclin dependent kinases (CDKs), which are typical serine/threonine kinases, work together with Cyclin B to drive the cell cycle forward [73]. The progression of cells through the G_1_, S, G_2_, and M phases is directly regulated by CDKs [74, 75]. P53 is activated to induce cell cycle stagnation when cells are subject to minor DNA damage for self-healing [76]. Cyclin B binds to CDK1 and is regulated by phosphorylation and dephosphorylation, which promotes the G_2_/M phase transition of cells [77]. It has been demonstrated that the expression of Cyclin B was up-regulated and the levels of ALT and AST were down-regulated after syr+cos administration, suggesting that syr+cos may activate the pathway related to liver regeneration, induce the expression of FXR target gene Cyclin B and promote liver repair in chemical liver injury [78], which was basically consistent with our research. In addition, it was reported that ALT/AST was related to the functional impairment progress of liver [79]. In the present study, the ratio of ALT/AST was lower in syr+cos treatment group than in LPS treatment group which indicating drug may significantly reduce the ratio with better prognosis. In present study, western blot results revealed that syr+cos might protect L-02 hepatocytes from LPS-induced ALI by increasing the expression of Cyclin B and CDK1 proteins.

TNF-α has been found to induce hepatocyte apoptosis, and its receptor plays a crucial role in liver injury caused by hepatotoxic drugs and sepsis, according to previous studies [80]. Furthermore, TNF-α expression was greatly reduced in mice with a knocked-out NF-κB gene [81], which when activated, triggers NF-κB translocation/transsituation from the cytomembrane to the cell nucleus, finally leading to the production of proteins such as p65 and IκBα, as well as IκBα phosphorylation. Inhibition of NF-κB signaling pathway had been proposed as the promising treatment for ALI, according to earlier experiments [82]. Syr+cos was found to interfere with the inflammatory mediators activated by LPS, which are controlled by the NF-κB signaling pathway [83]. Western blot analysis revealed that syr+cos may alleviate LPS-induced injury in L-02 hepatocytes by suppressing expression of NF-κB and TNF-α proteins within the NF-κB signaling pathway.

In the present study, we evaluated the hepatoprotective effect of syr+cos against LPS-induced ALI in L-02 cells. Consequently, the hepatoprotective effect of syr+cos on LPS induced ALI through the regulation NF-κB signaling pathway, apoptotic pathway and cell cycle. It was revealed that pretreated with syr+cos significantly promoting L-02 cell proliferation, inhibiting cell apoptosis and blocking cell cycle in G_1_ phase and G_2_/M phase respectively. It was also showed that pretreated with syr+cos significantly decreased the production of ALT, LDH, AST, MDA, and ROS while improved the levels of CAT, SOD respectively. Furthermore, syr+cos may downregulate the expression level of Caspase-3,7,9, NF-κB, TNF-α and upregulating the expression level of Cyclin B, CDK1 and p-IκB proteins. In addition, Rac1 transcriptional targets in the development of LPS induced L-02 cells were analyzed. Thus, by using short hairpin RNA (shRNA) approach, our study confirms that Rac-1 signaling is involved in ALI and that silencing of Rac-1 may protect the liver from ALI by increasing AKT, p-AKT (S473), p-AKT (T308) and reducing p-AKT proteins.

## CONCLUSIONS

Above that, syr+cos could be an effective candidate drug for the treatment of LPS-induced ALI via Rac1/AKT/NF-κB signaling pathway. The present study provided the experimental basis and data support for revealing the pharmacology mechanism of syr+cos on ALI.

## Supplementary Material

Supplementary Figures

Supplementary Files
