# Supplementary material for: Hepatoprotective effect of syringin combined with costunolide against LPS-induced acute liver injury in L-02 cells via Rac1/AKT/NF-κB signaling pathway
Source: Aging (Albany NY). 2023 Nov 1;15(21):11994–2020. doi: 10.18632/aging.205161 (PMC10683587; doi:10.18632/aging.205161)
Supplement: Supplementary Figures [file aging-15-205161-s001.pdf]

SUPPLEMENTARY FIGURES

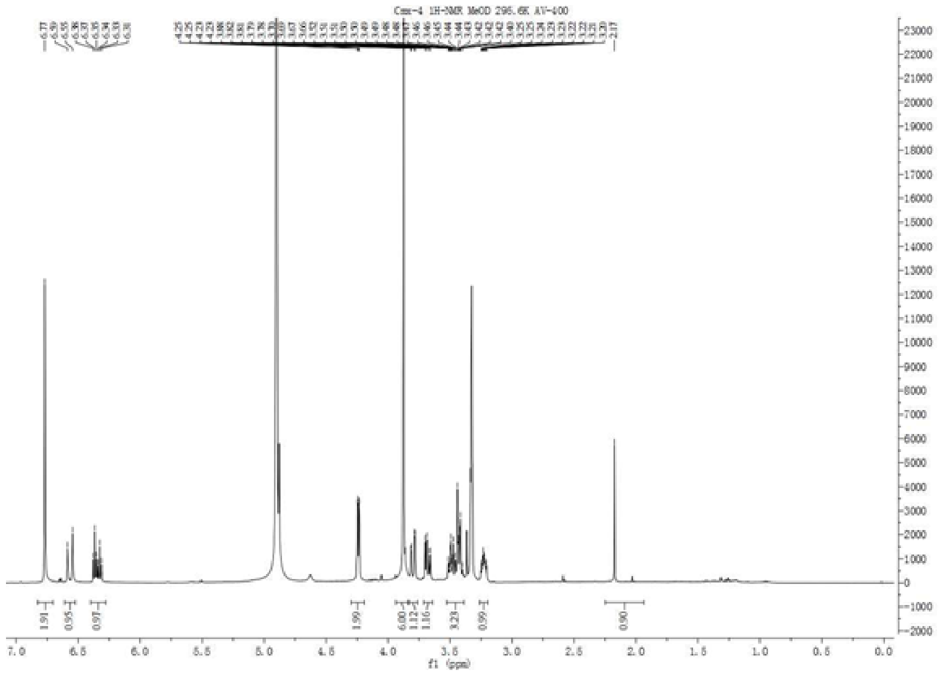

Supplementary Figure 1. The ^{1}H-NMR spectra of compound syringin.

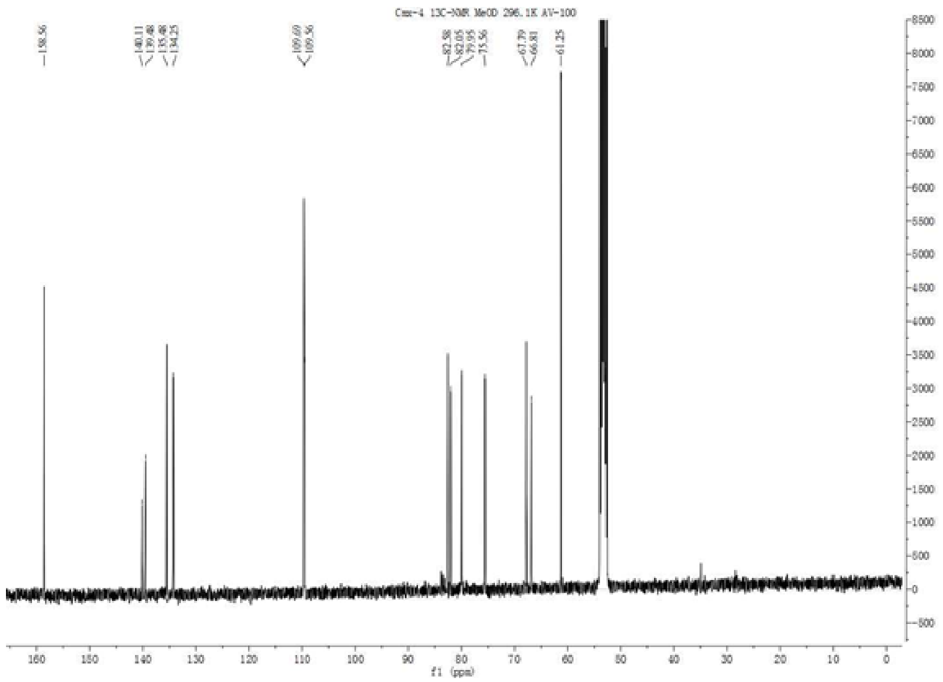

Supplementary Figure 2. The ^{13}C-NMR spectra of compound syringin.

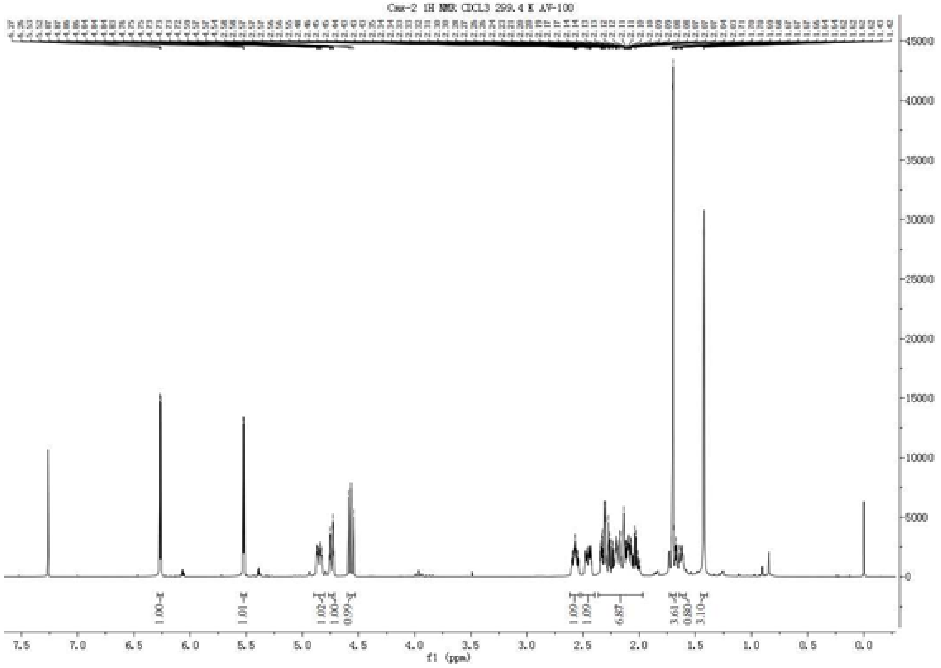

Supplementary Figure 3. The ^{1}H-NMR spectra of compound costunolide.

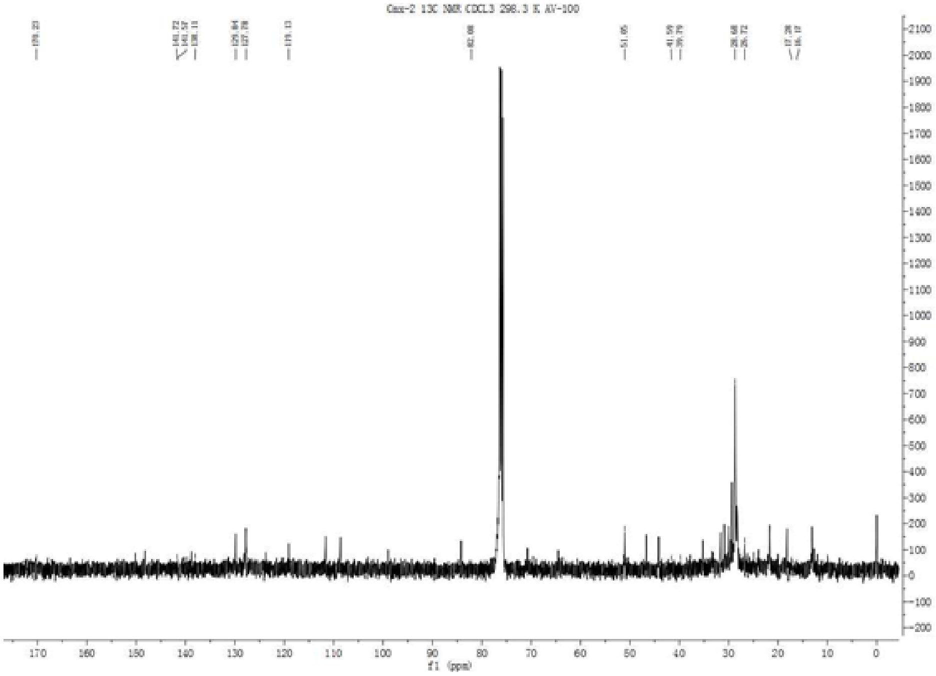

Supplementary Figure 4. The ^{13}C-NMR spectra of compound costunolide.

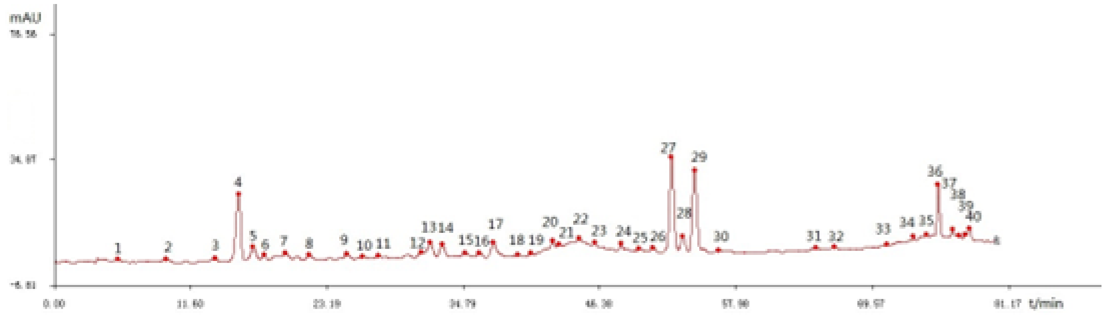

Supplementary Figure 5. The reference fingerprint of *Dolomiaea souliei* (Franch.) C.Shih.

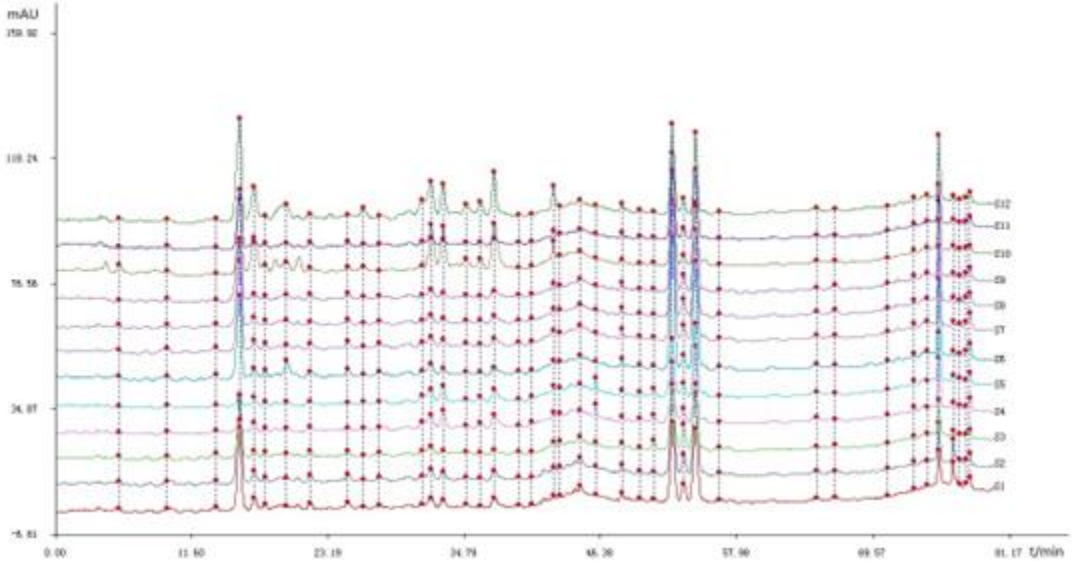

Supplementary Figure 6. HPLC fingerprints for 12 batches of *Dolomiaea souliei* (Franch.) C.Shih.
