# Supplementary material for: Hepatoprotective effect of syringin combined with costunolide against LPS-induced acute liver injury in L-02 cells via Rac1/AKT/NF-κB signaling pathway
Source: Aging (Albany NY). 2023 Nov 1;15(21):11994–2020. doi: 10.18632/aging.205161 (PMC10683587; doi:10.18632/aging.205161)
Supplement: Supplementary Files [file aging-15-205161-s002.pdf]

SUPPLEMENTARY FILES

Supplementary File 1. Chemical property of compound syringin.

ACS of syringin: 118-34-3

Syringin, white powder, molecular weight: 372.37, melting point: 174-177° C.

^{1}H-NMR (MeOD, 400 MHz) δ: 6.66 (2H, s, H-2, H-6), 6.45 (1H, d, *J* =15.9 Hz, H-7), 6.23 (1H, dt, *J* = 15.8, 5.5 Hz, H-8), 4.85 (1H, d, *J* = 7.8 Hz, H-1'), 4.13 (2H, dd, *J* = 5.6, 1.6 Hz, H-9), 3.80 (6H, s, 3,5-OCH_3), 3.76 (1H, dd, *J* = 12.0, 2.0 Hz, H-6'a), 3.56 (1H, dd, *J* = 12.0, 5.2 Hz, H-6'b), 3.33 (1H, m, H-2'), 3.37 (2H, m, H-4', H-5'), 3.20 (1H, m, H-3').

^{13}C-NMR (MeOD, 100 MHz) δ: 135.89 (C-1), 105.47 (C-2, C-6), 154.34 (C-3,5), 135.26 (C-4), 131.26 (C-7), 130.04 (C-8), 63.57 (C-9), 105.34 (C-1'), 75.73 (C-2'), 78.36 (C-3'), 71.34 (C-4'), 77.82 (C-5'), 62.59 (C-6'), 57.03 (3,5-OCH_3).

Supplementary File 2. Chemical property of compound costunolide.

ACS of costunolide: 553-21-9

Costunolide, white powder, molecular weight: 232.32, melting point: 106-107° C.

^{1}H-NMR ($CDCl_3$, 400 MHz) δ: 6.26 (1H, d, *J* =3.6 Hz, H-13a), 5.52 (1H, d, *J*=3.2 Hz, H-13b), 4.85 (1H, dd, *J* =11.6, 4.3Hz, H-1), 4.74 (1H, d, *J* =10 Hz, H-5), 4.57 (1H, t, *J* =9.9 Hz. H-6), 1.70 (3H, s, H-15), l.42 (3H, s, H-14) ^{13}C-NMR ($CDCl_3$, 100 MHz) δ: 127.78 (C-1), 28.68 (C-2), 41.59 (C-3), 141.57 (C-4), 129.84 (C-5), 82.08 (C-6), 51.05 (C-7), 26.72 (C-8), 39.79 (C-9), 138.11 (C-10), 141.72 (C-11), 170.23 (C-12), 119.13 (C-13), 16.17 (C-14) , 17.28 (C-15).
